# Chemistry and Hypoglycemic Activity of GPR119 Agonist ZB-16

**DOI:** 10.3389/fendo.2018.00543

**Published:** 2018-09-19

**Authors:** Ivan N. Tyurenkov, Denis V. Kurkin, Dmitry A. Bakulin, Elena V. Volotova, Evgeny I. Morkovin, Mikhail A. Chafeev, Ruben N. Karapetian

**Affiliations:** ^1^Volgograd State Medical University, Volgograd, Russia; ^2^Volgograd Medical Research Center, Volgograd, Russia; ^3^Chemical Diversity Research Institute, Khimki, Russia

**Keywords:** ZB-16, sitagliptin, T2D, hypoglycemia, GPR119 agonists, rats

## Abstract

This article is to highlight the chemical properties and primary pharmacology of novel GPR119 agonist ZB-16 and its analogs, which were rejected during the screening. Experiments were performed *in vitro* (specific activity, metabolism and cell toxicity) and *in vivo* (hypoglycemic activity and pharmacokinetics). ZB-16 exhibits nanomolar activity (EC50 = 7.3–9.7 nM) on target receptor GPR119 *in vitro* associated with hypoglycemic activity *in vivo*. In animals with streptozotocin-nicotinamide induced type 2 diabetes mellitus (STZ-NA T2D) daily oral dose of ZB-16 (1 mg/kg) or sitagliptin (10 mg/kg) for 28 days resulted in the reduction of blood glucose levels. The effects of ZB-16 were comparable to the hypoglycemic action of sitagliptin. ZB-16 demonstrated relatively low plasma exposition, high distribution volume, mild clearance and a prolonged half-life (more than 12 h). The present study demonstrates that the targeted search for selective GPR119 receptor agonists is a well-founded approach for developing novel drugs for the therapy of T2D. Based on the combination of high *in vitro* activity (compared to competitor standards), a useful ADME profile, distinct hypoglycemic activity which is comparable to the efficacy of sitagliptin in rats with experimental T2D, and the acceptable pharmacokinetic profile, we recommend the ZB-16 compound for further research.

## Introduction

Over the past century, there has been a dramatic increase in the prevalence of metabolic disorders such as diabetes mellitus and obesity. In accordance to International Diabetes Federation there are 425 million people with diabetes in the World, and this portion will increase up to 629 million in 2045 (1). About 12% of global health expenditure ($727 billion) is spent on diabetes, thus the development of novel medicines and treatment strategies plays crucial role in the improvement of the disease management. In the last few decades, there has been a surge of interest in the effects of incretins and their analogs on the glucose metabolism and utilization. Evidence suggests that G protein-coupled receptor 119 (GPR119) is implicated in the incretin-depended insulin synthesis and exocytosis (2, 3). Mostly expressed either on intestinal L- and K-cells or pancreatic β-cells, GPR119 had been validated as a potential target for novel pharmacological agents which could become a prominent medicine against type 2 diabetes mellitus (T2D), obesity and metabolic syndrome (4). The activation of GPR119 stimulates the secretion of incretins—glucagon-like peptide-1 (GLP-1) and glucose-dependent insulinotropic polypeptide (GIP) (5) are metabolic hormones which augment the secretion of insulin (GLP-1 and GIP) from pancreatic β-cells and inhibit glucagon release (GLP-1) in α-cells. Unlike GIP, the secretion of GLP-1 is impaired in patients with T2D (6, 7).

Both GLP-1 and GIP, being rapidly inactivated by the enzyme dipeptidyl peptidase-4 (DPP-4) expressed on the surface of most cell types, have relatively short life-time. Although several researches resulted in the development of synthetic GLP-1 analogs, resistant to the DPP-4 action, or DPP-4 inhibitors, extending the incretin action in T2D (8), the potency of GPR119 agonists to enhance the incretin action is not well studied (9, 10).

The 10 years of clinical experience shown that GLP-1 analogs and DPP-4 inhibitors exert moderate or potent hypoglycemic activity and are safe in patients with T2D. The secondary effects of GLP-1 analogs include the body weight decline and the decrease in risk of vascular complications of T2D. The main disadvantages are the lack of GLP-1 analogs for oral administration, or insufficient efficacy of DPP-4 inhibitors.

Previously we published our data (11) about the activity of novel GPR119 agonist ZB-16 *in vivo*, which exerted a potent anti-diabetic action in rat model of T2D. ZB-16 administered orally (1 mg/kg/day) for 28 day decreased the blood glucose levels under fasting conditions and improved the glucose utilization in male Wistar rats with streptozotocin-nicotinamide induced type 2 diabetes mellitus (STZ-NA T2D). These changes were associated with the increase of stimulated secretion of GLP-1 and insulin, accompanied by the growth of insulin-positive cells in pancreas. This article is to highlight the chemical properties and primary pharmacology of ZB-16 both *in vitro* and *in vivo* compared with other series of similar compounds rejected during the screening.

## Methods

### *In vitro* studies

#### Solubility evaluation

A Millipore filter system was used to measure the kinetic solubility in each of 3 Britton-Robinson universal buffer solutions of pH 2, 4, and 7 (0.04 M H_3_BO_3_, 0.04 M H_3_PO_4_ and 0.04 M CH_3_COOH titrated with 0.2 M NaOH) in the presence of 2% DMSO (12). Verapamil and diethylstilbestrol constituted the high and low solubility control solutions, respectively.

#### Specific activity *in vitro*

Specific activity was evaluated using an immortalized Chinese hamster ovary cell line CHO-K1 steadily expressed human GPR119 (hGPR119). The cells (10^7^ ml^−1^) were cultured in Dulbecco's modified Eagle medium (DMEM) modified with 10% fetal bovine serum (FBS), revolved continuously at 50 rpm at 37°C in 8% CO_2_ for 3–4 days. Lance Ultra cAMP kit (Perkin Elmer, Waltham, MA, USA) was chosen as the experimental platform (13). The experiments were performed in accordance to the manufacturer's instructions. The agonistic activity of the test compounds was estimated by the increase in intracellular cAMP concentration, assuming the hGPR119 activation is followed by the activation of cellular adenylate cyclase, which increases intracellular cAMP levels (4, 14). Known agonists of hGPR119 receptors were used as positive controls to determine the maximal possible effect (MPE). The activity of test compounds was expressed in terms of median effective concentration (EC_50_).

#### Biotransformation *in vitro*

The metabolism of the compounds was studied in commercially available microsomal fractions derived from human and rat hepatocytes. Test compounds (0.5 μM) were incubated with microsomal fractions in the presence of NADPH during 30 min in a shaker at 37°C. Reactions were stopped at 0, 5, 10, 15, and 30 min by adding acetonitrile. After proteins precipitation, the remaining amount of the test compound in the supernatant was detected with high performance liquid chromatography tandem-mass spectrometry (HPLC-MS/MS) method using Agilent 1260 liquid chromatograph (Agilent Technologies, Santa Clara, CA, USA) and a QTRAP6500 triple quadrupole mass spectrometer with a TurboIonSpray electro-spraying module (ABSciex, Foster City, CA, USA) (15). Incubation was performed twice, with at least 2 measurements for each replicate (*n* ≥ 3). The half-life (T_1/2_), the clearance *in vitro* (CL_int_) and the remaining amount of substance (% of initial quantity) were calculated.

Inhibition of cytochrome P450 (CYP) isozymes, in particular CYP3A4, CYP1A2, CYP2C9, CYP2C19, and CYP2D6, was studied using Vivid® CYP450 Screening Kits (Invitrogen Corporation, Carlsbad, CA, USA) in accordance to the Invitrogen protocol (Vivid CYP450 screening kit protocol O-13873-r1 US 0405) in a 384-well plate format. Ketoconazole, α-naphtoflavone, sulfaphenazole, miconazole and quinidine (Sigma-Aldrich) were used as the control inhibitors of CYP3A4, CYP1A2, CYP2C9, CYP2C19 and CYP2D6 correspondingly (16). Test compounds were incubated for 15 min in the presence of a NADPH-regenerating system. Briefly, the reaction was initiated by adding NADP^+^ and a substrate converted to fluorescent derivates by specific CYP isozyme. The reaction was terminated by adding 1M Tris. The fluorescence of the reaction product, which was proportional to CYP isozyme activity, was measured. Test compounds and the control inhibitor were tested twice at concentrations ranging from 0.0046 and 10 μM. The maximal enzyme activity determined in 1% DMSO with no compounds added. The minimal signal was defined as the signal obtained without enzyme incubation. The inhibitory action of test compounds on the CYP isozymes was expressed in terms of median inhibitory concentration (IC_50_).

#### Cell toxicity *in vitro*

Cell viability was estimated by the quantity of ATP produced by the HepG2 cell culture, derived from human liver hepatocellular carcinoma. Experiments were performed using a Cell Titer Glo Cell Viability (Promega) test system (17). Tubercidin, an inhibitor of various cellular metabolic processes, including RNA processing, nucleic acid synthesis, protein synthesis, and methylation of tRNA through intracellular incorporation into nucleic acids, was used as a positive control (18). Median inhibitory concentration (IC_50_) was used as a quantitative parameter to evaluate cell toxicity. To evaluate inhibition effectiveness (% Inh), the following formula was used: % Inh = [(L_pos_ − L_exp_)/(L_pos_ − L_neg_)] ^*^ 100%, where L_pos_ is the positive control (luminescence in cells with no compound); L_neg_ is the negative control (luminescence in cells containing medium but no cells); and L_exp_ is the luminescence in cells containing the compound at a certain concentration. IC_50_ values were calculated using Prism 5 and the criteria for minimization of the experimental points squares deviation from the theoretically calculated curve algorithm.

Cardiac toxicity was evaluated by estimating the influence of each substance on hERG potassium channels using the *in vitro* test system Invitrogen® PredictorTM hERG (Invitrogen Corporation, Carlsbad, CA, USA), containing Predictor hERG membrane preparations, fluorescent tracer stock solution and an assay buffer solution. Experimental procedures were conducted in accordance with the PredictorTM hERG test system's manufacturer instructions. Assays were performed in 384-well microplates. Each well contained 5 μL aliquot of each concentration of test compound, 10 μL of (2X) membrane preparation and 5 μL of working tracer solution. The cumulative reaction volume was 20 μl with 1% of DMSO (19). All procedures were performed at 4 replicates for each concentration. The plate was allowed to incubate at room temperature for at least 1 h prior to fluorescence polarization measurements performed with a microplate reader Infinite M1000 PRO (Tecan).

### *In vivo* studies

All animal experiments were conducted in accordance with ethical animal research standards defined by Russian law (20), Guidelines for preclinical trials of medicinal products (21), Guide for the care and use of laboratory animals (22) and approved by the local ethical committee (VMRC, Volgograd, Russian Federation; registration number IRB 00005839 IORG 0004900 (OHRP), Protocol Number: 191-2014, February 25, 2014). The present study used female Wistar rats aged 3–4 months weighing 250–280 g. Animals were housed 4–6 per cage in a separate room with controlled environment (20–26°C, relative humidity 50–70%, consistent 12/12 light/dark regimen, 8:00 lights on) with food and water *ad libitum* (except intentional fasting needed for blood glucose levels determination described below). At the end of the experiment animals were sacrificed by an overdose of chloral hydrate (800 mg/kg).

#### Evaluation *in vivo* of the hypoglycemic action in intact rats

Blood glucose levels were evaluated in the peripheral blood (from the tail vein) of intact fasted (6 h) female Wistar rats using a glucometer Contour TS (Bayer, Germany) at 60, 120, and 180 min after single oral administration of each ZB-09 analogs (in doses of 0.1, 1, and 10 mg/kg). The female animals were used because of their advantages in toxicological studies shown by several authors (23).

#### Evaluation of the hypoglycemic action in a rat model of T2D

Procedures were performed in 40 female Wistar rats weighing 250–280 g. Streptozotocin-nicotinamide-induced rat model of T2D was used (24, 25). Briefly, streptozotocin (Sigma, St. Louis, MO, USA) was administered i.p. to overnight fasted rats (65 mg/kg in 100 mmol/l citrate buffer, Ph 4.5). Nicotinamide (Sigma, St. Louis, MO, USA) (230 mg/kg) dissolved in saline was administered i.p. 15 min before streptozotocin. STZ-NA T2D had been confirmed 72 h after in rats with blood glucose levels in a range 9–14 mmol/l measured in fasting (6 h) conditions. Animals failed to reach the criteria of STZ-NA T2D were excluded from the experiment. Following the continuous oral administration of sitagliptin (10 mg/kg), ZB-16 (1 mg/kg) or saline during 28 days STZ-NA T2D confirmation the fasting blood glucose levels were measured, and oral glucose tolerance test (OGTT) was performed. For OGTT rats received an oral dose of 3 mg/kg glucose dissolved in saline; blood glucose levels were monitored every 30 min for 2 h after glucose administration. Sitagliptin dose was chosen in accordance with published data (26).

#### Evaluation of pharmacokinetics *in vivo*

The pharmacokinetics of ZB-16 in a dose of 10 mg/kg after single oral or intravenous administration was studied in 6 male Wistar rats weighing 250–280 g. The blood from the tail vein (200 μL) was collected before and at 0, 0.25, 0.5, 1, 2, 4, 8, 12, and 24 h after ZB-16 administration. The blood concentrations of the test compounds in animals were evaluated using HPLC-MS/MS. Basic pharmacokinetic parameters were calculated using a non-model method and WinNonlin Professional 6.0 (Pharsight Corporation, Mountain View, CA, USA) based on the experimental concentration-time data obtained on each animal. The list of calculated parameters included: AUC_0 → *t*_ (ng ^*^ hour/ml)—area under the “drug concentration—time” curve from the injection until the last measurement; AUC_0 → ∞_ (ng ^*^ hour/ml)—area under the curve from the injection until infinity; C_max_ (ng/ml)—maximal blood plasma drug concentration; C_0_ (ng/ml)—calculated initial blood plasma concentration of the drug after i.v. injection; k_el_ (1/hour)—velocity of elimination constant (the parameter that characterizes the velocity of elimination of the compound from blood plasma); MRT (hour)—average compound lifetime in the organism from the moment of injection; t_max_ (h)—time required for the compound to achieve its maximum blood plasma concentration; *t*_1/2_ (h)—half-life period (the time required for half of the compound to be eliminated from the blood plasma); F (%)—relative bioavailability, calculated using the following formula: F (%) = 100% ^*^ [AUC_0 → *t, p*.*o*._^*^ D_i.v._]/[AUC_0 → *t, i*.*v*._^*^ D_p.o._], where D – p.o. or i.v. dose.

### Statistical analysis

Statistical analysis was performed using GraphPad Prism 5.0 (GraphPad Software, San Diego, CA, USA). The distribution of all data was estimated using the Shapiro-Wilk normality test. Kruskal-Wallis test with Dunn's *post hoc* test was used for non-parametric data distribution. One-way ANOVA or repeated measures two-way ANOVA with Newman-Keuls *post hoc* test was performed for data sets obtained from Gaussian distribution (indicated in the figure legends).

## Results

### Development and screening

**Figure 1:**
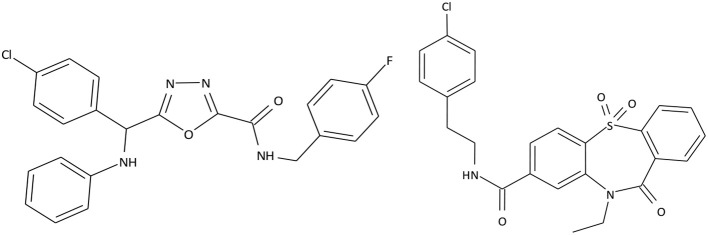
Structures of C301-5947 and C530-0315.

Based on selective high throughput screening of the data library consisting of 1.5 million small organic molecules, 2 prospective compound series were chosen. The structures of the most promising compounds from these series are presented in Figure 1.

C301-5947, C530-0315, and 40 similar compounds were synthesized. Each compound was screened for primary agonist activity (EC_50_) to evaluate putative structure-effect patterns and to further target structure modifications. Activity screening results are presented in Supplement 1. Non-active compounds are shown at Supplement 2.

**Figure 2:**
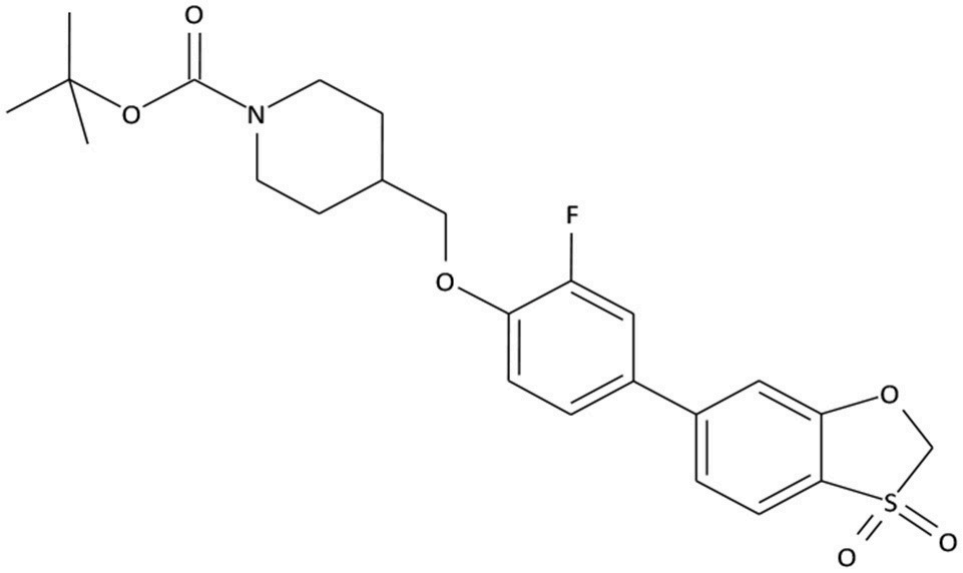
Primary structure of ZB-09.

The compounds exhibiting low levels of activity compared with Arena (0119) we rejected from further investigation. After analyzing the open access formulas of compounds synthesized by pharmaceutical companies and introducing new and original elements into their structures, ZB series of test compounds was developed (Figure 2).

ZB-09 exhibited an EC_50_ = 4 nM, comparable to the standard compound Arena (0119). At this stage, **absorption**, **distribution**, **metabolism** and **excretion** parameters (ADME) were evaluated. The test compound ZB-09 was found to be barely soluble in water at all pH levels investigated, significantly limiting its further use (Table 1).

**Table 1:** Solubility of ZB-09 by pH.

**Compound**	**Solubility (**μ**M) UB, 2% DMSO**		
	**pH 2**	**pH 4**	**pH 7**
ZB-09	<3	<3	<3
Diethylstilbestrol	12	10	12
Verapamil	212	203	190

The biotransformation assay revealed that ZB-09 exhibits a high rate of microsomal oxidation (Table 2), which can lead to inferior pharmacokinetics *in vivo*, including the short life time and low blood concentrations of the compound. Therefore, we focused on improving the properties of the compound by modifying its structure. First, we had to increase the solubility and microsomal stability of the compound.

**Table 2:** Microsomal stability of ZB-09.

**Microsomal fraction**	***t*_1/2_, min**	**CL_int_**	**CL_int, hep_**	**CL_h_**	**ER**
Human	4.16	0.6664	770.6916	20.44	0.94
Rat	31.08	0.0892	160.56	40.97	0.74
Mouse	8.57	0.3236	1274.175	84.06	0.93

The structures and activities of the synthesized analogs of ZB-09 are summarized in Table 3.

**Table 3:** Structures and activities of ZB-09 analogs.

**ID**	**Structure**	**C_max_, μM**	**EC_50_, M**
Arena (0119)		10	1.19 * 10^−8^
ZB-17	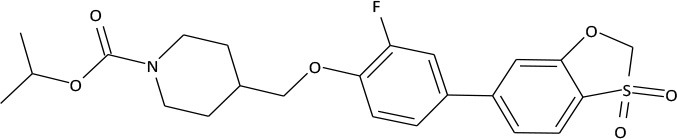	30	1.25 * 10^−8^
ZB-16	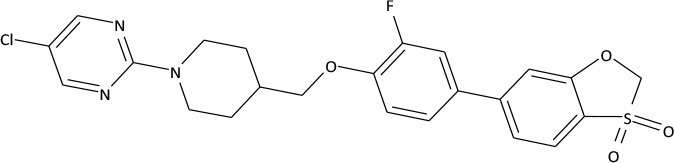	30	7.25 * 10^−9^
ZB-18	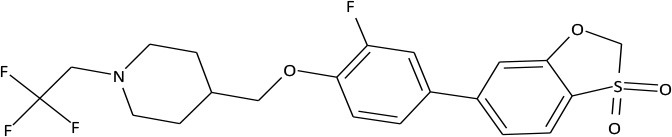	30	6.35 * 10^−8^
ZB-19	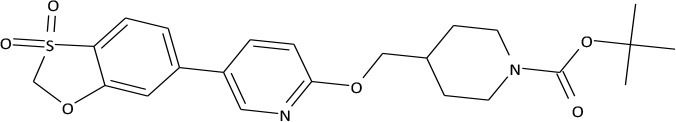	30	1.1 * 10^−8^
ZB-20	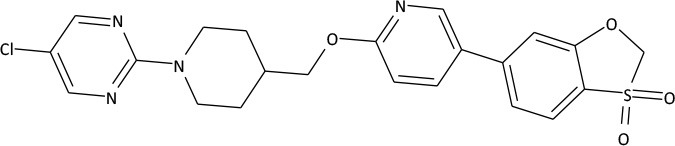	30	2.93 * 10^−8^

ZB-09 analogs showed the activity levels within the Arena (0119) standard range. Table 4 presents pH-depended solubility of these compounds.

**Table 4:** Effects of pH on the water solubility of ZB-09 analogs.

**Tested compound**	**Solubility (**μ**M) UB, 2% DMSO**		
	**pH 2**	**pH 4**	**pH 7**
Diethylstilbestrol	15	14	14
Verapamil	203	200	191
ZB-18	25	<3	<3
ZB-16	52	<3	4
ZB-17	<3	<3	<3
ZB-19	<3	<3	<3
ZB-20	<3	<3	<3

ZB-16 was found to be the most effective compound investigated. Nonetheless, we decided to further investigate ZB-19 and ZB-20 due to their high agonistic activity.

#### Microsomal stability of ZB-09 analogs

As seen in Table 5, ZB-16 exhibits significantly higher resistance in human and rat microsomes compared to ZB-09, the primary compound of the series.

**Table 5:** Microsomal stability of test compounds.

**Compound**	**Microsomal fraction**	***t*_1/2_, min**	**CL_int_**	**CL_int, hep_**	**CL, h**	**ER**
ZB-16	Human	42.52	0.0652	75.4038	16.43	0.76
	Rat	64.77	0.0428	77.04	32.09	0.58
ZB-19	Human	2.50	1.1076	1280.939	20.66	0.95
	Rat	21.93	0.1264	227.52	44.29	0.81
ZB-20	Human	40.76	0.068	78.642	16.57	0.76
	Rat	130.75	0.0212	38.16	22.53	0.41

The ZB-19 compound is the most similar to the standard compound Arena (0119) with regard to *in vitro* activity, although the presence of the end tert-butyl-oxycarbonyl group makes ZB-19 nonresistant to liver enzymes. The ZB-20 compound, in which, as for ZB-16, the tert-butyl-oxycarbonyl group was replaced with a resistant heterocyclic bioisostere, chlorine-pyrimidine, did not exhibit increased solubility in water (as was the case with ZB-16) but did exhibit increased resistance to liver enzymes. This compound has an *in vitro* EC_50_ of 29 nM, which is less than those of the ZB-16 compound and the Arena (0119) but still acceptable.

#### Cardiac toxicity of leader compounds

The human Ether-à-go-go-Related Gene (hERG) codes the alpha subunit of a potassium ion channel which contributes to the electrical activity of the heart. Inhibition or compromising of activity of this ion channel could result in long QT syndrome creating the concomitant sudden death risk (27). Results obtained with regard to the binding of compounds from the ZB series to hERG are presented in Table 6.

**Table 6:** Binding of compounds from the ZB series to hERG.

**Test compound**	**Concentration, μM**	**IC_50_, M**	**LogIC_50_**	**Average polarization mP (*n* = 4)**	**SD**	**CV%**	**Unbound hERG %**
Standard	4	2.28 * 10^−8^	−7.6	–	–	–	–
ZB-16	10	–	–	244	4.5	1.8	77
ZB-16	1	–	–	264	8.7	3.3	89
ZB-20	10	–	–	263	4.6	1.7	91
ZB-20	1	–	–	261	4	2	89

The test compounds bind to less than 50% hERG in both concentrations tested (10 and 1 μM). This results in minimal cardiac toxicity risk in regard to the activity of hERG ion channels.

#### Cell toxicity of leader compounds

**Figure 3:**
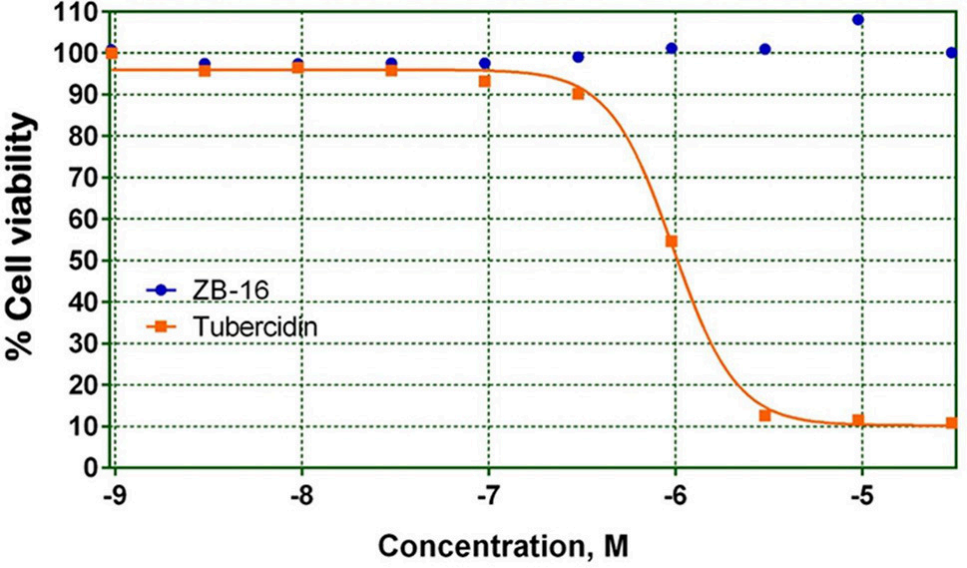
HepG2 cell culture viability on the presence of test compound ZB-16 or the control compound Tubercidin. The data expressed as the means (*n* ≥ 3); the response was normalized to the values of the cells cultured without test or control compounds.

As shown in Figure 3 ZB-16 had no effect on HepG2 cell culture viability.

#### CYP450 inhibition by leader compounds

Parameters pertaining to CYP450 inhibition by ZB series are presented in Table 7.

**Table 7:** Inhibition of CYP450 isoforms by ZB-16 and ZB-20 test compounds and standard compounds.

**Test compound**	**CYP2C9**	**CYP3A4**	**CYP1A2**	**CYP2C19**	**CYP2D6**
ZB-16	>1 * 10^−5^	>1 * 10^−5^	>1 * 10^−5^	>1 * 10^−5^	>1 * 10^−5^
ZB-20	>1 * 10^−5^	>1 * 10^−5^	>1 * 10^−5^	>1 * 10^−5^	>1 * 10^−5^
The reference inhibitor	Sulfaphenazole	Ketoconazole	α- Naphthoflavone	Miconazole	Quinidine
Ref. inh. IC_50_ Ref. inh. IC_50_ (Invitrogen)	3.5 * 10^−7^ 2.1 * 10^−7^	6.9 * 10^−8^ 7 * 10^−8^	1.6 * 10^−8^ 3 * 10^−8^	2.9 * 10^−8^ 4 * 10^−8^	3.0 * 10^−9^ 10 * 10^−9^

As seen in Table 7, ZB-16 and ZB-20 inhibit neither of the CYP450 isoforms, indicating that the metabolism of other drugs is unlikely to be affected.

#### GPR119 agonistic activity of leader compound

**Figure 4:**
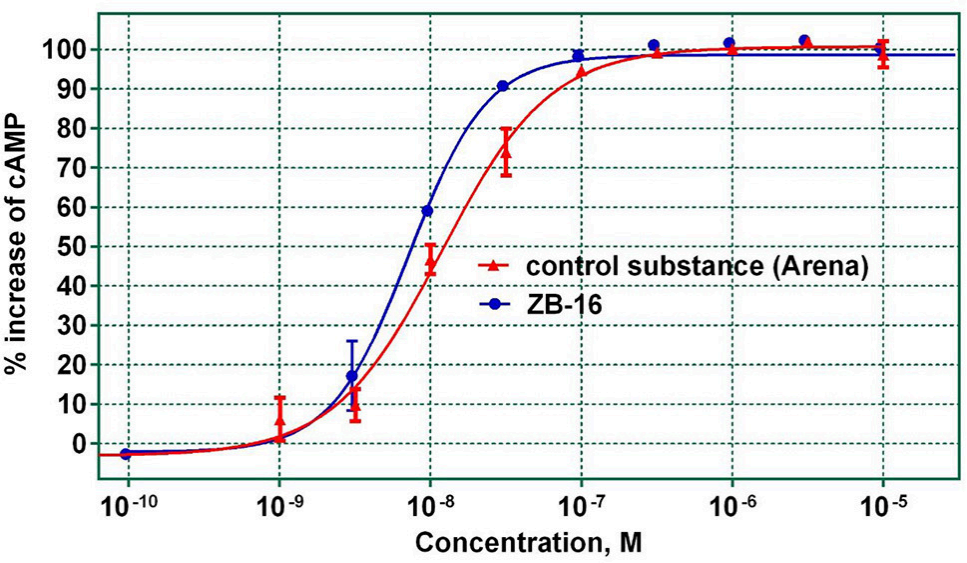
The agonistic activity of ZB-16 and the control substance (Arena) with regard to hGPR119 target receptors. The data expressed as the mean ± SEM (*n* ≥ 3); the response was normalized to the maximal response value obtained with the control substance.

The relationship between GPR119 agonistic activity and concentration was analyzed for the ZB-16 compound. The EC_50_ was calculated from the graph shown in Figure 4.

As seen in Figure 4, ZB-16 exhibits nanomolar activity (EC_50_ = 7.3–9.7 nM) on target GPR119 receptors, indicating that ZB-16 is a complete agonist. Based on the determined ADME parameter set and high activity *in vitro*, ZB-16 and ZB-20 were selected for *in vivo* studies.

### Hypoglycemic activity of leader compounds

**Figure 5:**
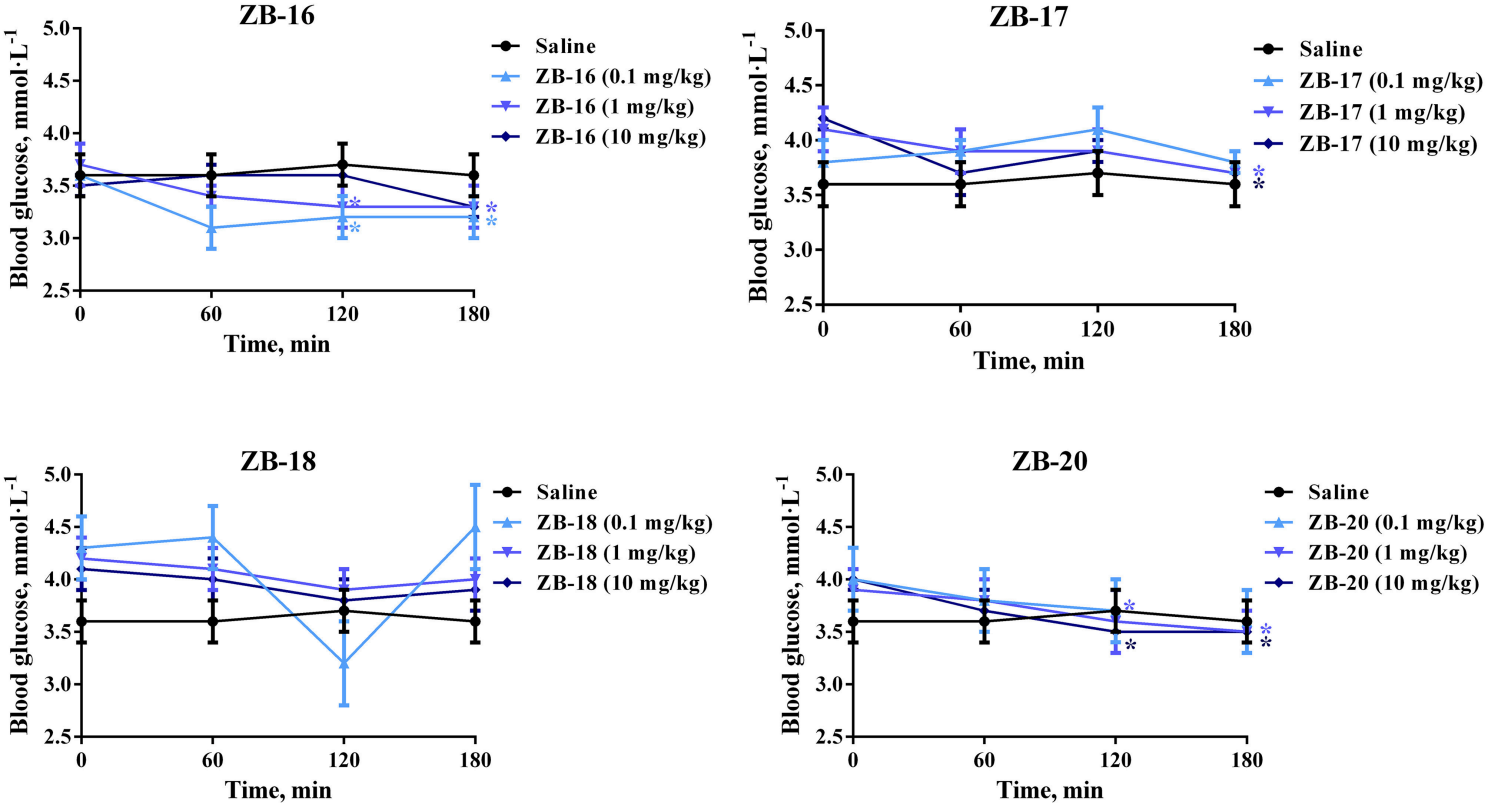
Hypoglycemic action of ZB-09 analogs in intact rats after single oral administration. The data expressed as the mean ± SEM (*n* = 10); *p < 0.05, significant, compared to initial levels of glycemia (2-way ANOVA with Newman-Keuls *post hoc*); no significant difference was seen at 0 min (*p* > 0.05).

ZB-09 analogs were tested *in vivo* for their hypoglycemic action in intact rats. All compounds, except ZB-19, were included in this study. The *in vivo* results are presented in Figure 5.

ZB-16 caused significant decrease in blood glucose levels after single oral administration of 0.1–1 mg/kg. ZB-17 and ZB-20 both exhibited similar hypoglycemic activity at higher dosages (1–10 mg/kg).

#### Hypoglycemic activity of ZB-16 after single oral administration in T2D rat model

**Figure 6:**
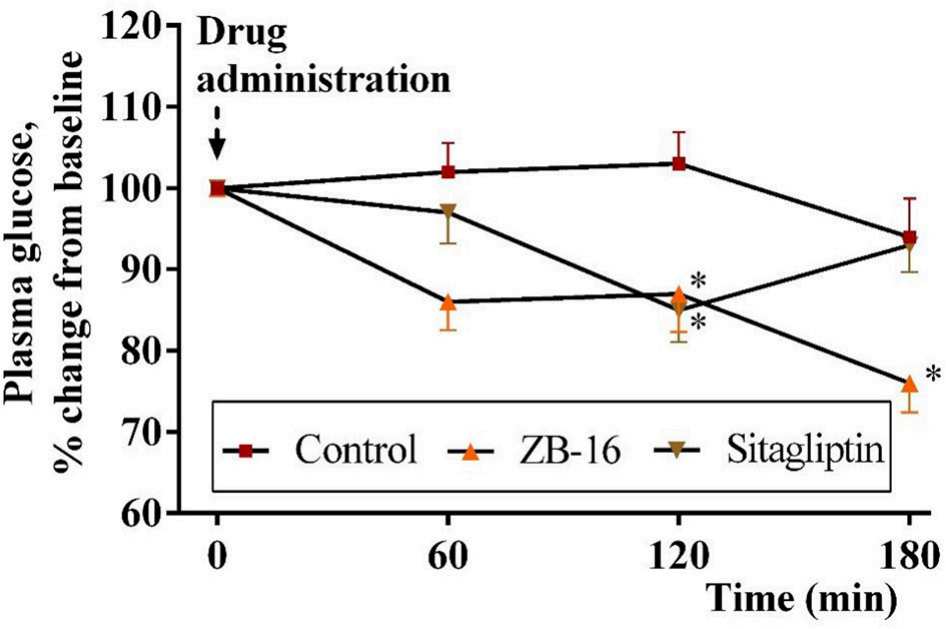
The decrease in plasma glucose level (changes from baseline) in rats with experimental diabetes over a 3 h period after single intragastral administration of ZB-16 or sitagliptin. The data were normalized to initial values and are expressed as the mean ± SEM (*n* = 10); Intact, the intact group of animals; Control, group of non-treated animals with experimental diabetes; *p < 0.05, significant, compared to the Control group (2-way ANOVA with Newman-Keuls *post hoc*).

Single oral administration of ZB-16 (1 mg/kg) in fasted rats 3 days after the confirmation of streptozotocin-nicotinamide (STZ-NA) induced T2D caused a 24% decrease of blood glucose levels over a 3 h period. This hypoglycemic effect was more prominent than that obtained for sitagliptin (10 mg/kg) (*p* < 0.05). This effect increased over time and continued until the end of the observation period (Figure 6).

#### Hypoglycemic activity of ZB-16 after continuous oral administration in T2D rat model

The animals received daily oral dose of ZB-16 (1 mg/kg) or sitagliptin (10 mg/kg) for 28 days after the confirmation of STZ-NA induced T2D. Body weight of rats are shown at Supplement 3. On 28 day of treatment oral glucose tolerance test was conducted.

**Figure 7:**
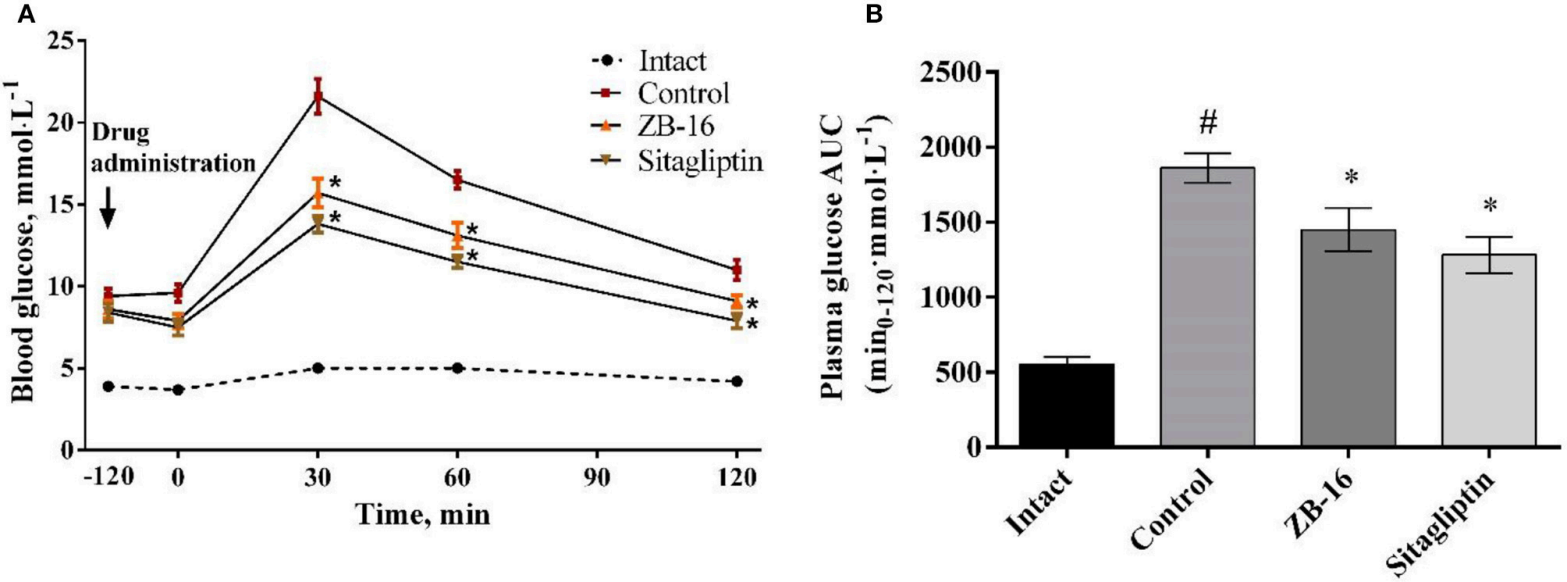
Blood glucose levels during oral glucose tolerance test **(A)**, and the area under the “glucose concentration-time” **(B)** in rats with T2D, obtained after 28 days of treatment. The data expressed as the mean ± SEM (*n* = 10); Intact, intact group of animals; Control, group of animals with experimental diabetes without treatment; *p < 0.05, significant, compared to the Control group; #*p* < 0.05 – compared to the Intact group (2-way ANOVA with Newman-Keuls *post-hoc*).

No significant changes were observed in control animals treated with saline. ZB-16 group showed blood glucose levels by 27% lower than in saline group 30 min after oral glucose administration (3 mg/kg). One hour after glucose administration, glucose blood levels were slightly lower in the treated groups compared to the control group. Two hours after glucose administration the mean blood glucose level in rats received ZB-16 was 9.1 ± 0.37 mmol/l, which was significantly lower than in the control group (Figure 7A).

Compared to the control group 22% reduction in the area under the “glucose concentration-time” curve was observed in ZB-16 group (Figure 7B). The effects of ZB-16 were comparable to the hypoglycemic action of sitagliptin.

#### Pharmacokinetic parameters of leader compounds

The pharmacokinetics of ZB-16 was studied in rats after single intravenous or oral administration in a dose of 10 mg/kg (Tables 8, 9).

**Table 8:** Pharmacokinetic parameters of ZB-16 in rats after i.v. injection (*n* = 3).

**Rat #**	***t*_1/2_**	**C_0_**	**AUC _0-t_**	**AUC _0−∞_**	**K_el_**	**V_z_**	**CL**	**MRT last**
	**h**	**ng/ml**	h*ng/ml	h*ng/ml	**1/h**	**l/kg**	**l/h/kg**	**h**
1	21	29	226	441	0.03	139	5	10
2	11	26	184	247	0.06	127	8	8
3	12	42	244	329	0.06	104	6	8
Mean ± SD	15 ± 5.8	32 ± 8.7	218 ± 30.4	339 ± 97.2	0.05 ± 0.017	123 ± 18.1	6 ± 1.8	9 ± 1.0

**Table 9:** Pharmacokinetic parameters of ZB-16 in rats after oral administration (*n* = 3).

**Rat #**	***t*_1/2_**	**C_max_**	**t_max_**	**AUC _0-t_**	**AUC _0−∞_**	**K_el_**	**MRT last**
	**H**	**ng/ml**	**h**	h*ng/ml	h*ng/ml	**1/h**	**h**
1	19	4	4	69	124	0.04	10
2	10	7	4	104	128	0.07	9
3	10	6	4	83	105	0.07	9
Mean ± SD	13 ± 5.2	6 ± 1.2	4 ± 0.0	85 ± 17.6	119 ± 11.9	0.06 ± 0.019	9 ± 1.0

ZB-16 demonstrated relatively low plasma exposition, high distribution volume, mild clearance and a prolonged half-life (more than 12 h). The median absorption time (MAT) of ZB-16 was calculated using average MRT values and the following formula: MAT = MRT_p.o._ − MRT_i.v._. The absolute bioavailability of the ZB-16 was 8%.

## Discussion

Diabetes mellitus (DM) is increasingly recognized as a worldwide public health concern. The prevalence of DM has experienced unprecedented growth over the past years. In 1998, King et al. (28) predicted an increase in patients with DM from 135 to 300 million by 2025. Although this prediction seemed to be quite pessimistic, the current population of people affected by DM is foundto reach 425 million (1).

The main challenge faced by many researchers is the lack of effective treatment for T2D due to various etiological, pathogenetic and social causes resulting from the individual characteristics of the course of the disease in the patient and his/her lifestyle (1). However the list of anti-diabetic medications, which are the major way to establish the T2D management, is continuously updating. To date, standards of medical care in diabetes include such pharmacological groups as secretagogues (sulfonylureas), sensitizers (biguanides, thiazolidinediones), glycosurics (inhibitors of the sodium glucose cotransporter type 2), alpha-glucosidase inhibitors, and peptide analogs (GLP-1 analogs and agonists or DPP-4 inhibitors), as well as human or recombinant insulin (29).

In recent years, there has been a growing number of publications focusing on the problem of exogenous influence on the incretin system (30–32). Incretins are gastrointestinal-derived hormones released in response to a meal playing a key role in the regulation of postprandial secretion of insulin and glucagon by the pancreas (33). The interest in the incretin system is based on the fact that the incretin effect is severely reduced or absent in patients with T2D (34). Thus, the restoration of adequate incretin biosynthesis and metabolism could become a promising treatment strategy for T2D (35). In particular, this approach includes the development of drugs able to stimulate the incretin secretion via activation of GPCR, found on the intestinal enteroendocrine cells. This group of receptors acts as the sensors of fatty acids, their derivates and some other digestion products. The activation of such receptors leads to the stimulation of incretin secretion, which, in turn, stimulates the synthesis and secretion of insulin, providing a state of postprandial normoglycemia (36, 37). In particular, the activation of GPR119, expressed in L- and K-cells of intestine as well as in pancreatic β-cells (38), leads to glucose-depended activation of insulin secretion (4, 5). Such mechanism of GPR119 agonists action is proposed to be beneficial because it could provide a pronounced antihyperglycemic effect in T2D without the risk of excess hypoglycemia. Therefore, these substances are considered as promising candidates for the role of drugs for T2D treatment (9).

Previous non-clinical studies and investigations performed in healthy volunteers has established that GPR119 are able to increase the level of circulating incretins including GLP-1, GIP, and tyrosine-tyrosine peptide (PYY), reducing the hyperglycemia after oral glucose load (39). Furthermore, animal studies demonstrated several secondary pharmacodynamic effects including cerebral, cardiac and endothelial protection which are in contrast to the anti-diabetic medications, which have only the hupoglycemic action. These secondary (or “pleiotropic”) effects could make GPR119 agonists essential for the prevention of T2D complications (40, 41).

Following the trends described above this work was to elucidate the particular steps of early non-clinical development of GPR119 agonists as a potential oral hypoglycemic drugs for human use.

The synthesis of test compounds was based on the results of computer modeling. Following this step the most active structure—ZB-09 was developed and consequentially modified to improve its ADME parameters. A highly-lipophilic tert-butoxycarbonyl group was found metabolically unstable. Furthermore, it was chemically unstable, particularly in acid medium, which limits oral bioavailability. Thus, the bioisostere of the tert-butoxycarbonyl group must meet requirements related to size, electronic density, polar distribution and solubility.

The synthesis of ZB-17 was performed by replacement of the tert-butoxycarbonyl group by other urethane fragment—an isopropyl-oxycarbonyl group.

The nonclassical bioisostere of the tert-butoxycarbonyl group is a heterocyclic aromatic structure, where nitrogen atoms replace the oxygen atoms of oxycarbonyl and additional modifications alter the lipophilicity. In the resulting ZB-16 compound, chloropyrimidine replaces urethane near the nitrogen.

We also attempted to change the size of the replacement group without changing its lipophilicity and polarity, which appeared to be possible due to the replacement of the tert-butoxycarbonyl group with a trifluoroethyl group. In this case, the additional fluorine atoms on the aliphatic fragment play a double role: first of all, they modify lipophilicity (more fluorine atoms means higher lipophilicity); secondly, due to their electronegativity, they increase the electrophilic effect of the replacement, bringing the “electronic action” of the alkyl replacement close to that of the oxycarbonyl replacement (i.e., the nitrogen in this group is not basic like the alkyl group). Therefore, the ZB-18 structure was introduced.

The presence of a highly lipophilic fluorine-phenyl structure fragment contributes to the low solubility of the end compound in aqueous solutions. The phenyl bioisostere with slightly higher hydrophilicity is pyridine due to the potential formation of hydrogen bonds near nitrogen atom. Likewise, a ZB-19 compound was designed in which fluorine-phenyl was replaced with pyridine. The ending tert-butyl-oxycarbonyl group, which is primarily responsible for the low microsomal stability, was replaced with chlorine-pyrimidine in the ZB-20 compound.

The data obtained *in vitro* indicate that all the ZB-09 analogs synthesized have activities in the Arena (0119) standard range. Replacement of the tert-butyl-oxycarbonyl group with its possible bioisosteres did not lead to significant loss of activity (except for a slight decrease in activity in the case of the trifluoroethyl group).

The solubility data obtained for the compounds synthesized indicate that replacing the tert-butyl-oxycarbonyl group to an isopropyl-oxy-carbonyl group did not increase the water solubility in solutions of any pH studied. However, replacing this group on the trifluoroethyl group, and especially on chloropyrimidine, increased solubility in acidic media (pH 2). This may improve the compound's pharmacokinetics and stomach absorption. Therefore, due to its solubility, ZB-16 is the most effective compound investigated. Nonetheless, we decided to further investigate 2 compounds from the series (ZB-19 and ZB-20) due to their high agonistic activity.

ZB-16 exhibits significantly higher resistance in human and rat microsomes compared to ZB-09, the primary compound of the series. Thus, bioisostere replacement of the tert-butyl-oxycarbonyl group with a nonclassical bioisostere such as chloropyrimidine enabled us to increase both problematic parameters of the series: low water solubility (which increases as the pH decreases) and microsomal stability.

ZB-16 exerted nanomolar agonist activity on human GPR119 receptors associated with the absence of cytotoxic effects in standard tests for cellular toxicity. The study of the hypoglycemic activity of ZB-16 in intact animals and animals with experimental diabetes in cases of single and chronic (4 weeks) administration, revealed an extensive hypoglycemic effect and decreased glucose tolerance as assessed by oral glucose tolerance test. This action is associated with a previously reported increase of GLP-1 release. Thus, this study produced results which are consistent with the findings of a great deal of our previous work on the chronic oral administration of ZB-16 to male rats with STZ-NA-induced T2D, demonstrated that ZB-16 exerts a pronounced antidiabetic activity due to the improvement of glucose utilization by stimulating GLP-1 and insulin secretion, as well as protective action on pancreatic β-cells (11).

In conclusion, the present study demonstrates that the targeted search for selective GPR119 receptor agonists is a valid approach for developing novel drugs for the therapy of T2D. Based on the data presented, it can be argued that the GPR119 agonists have a high therapeutic potential. High *in vitro* activity (compared to competitor standards), a useful ADME profile, distinct hypoglycemic activity comparable to the hypoglycemic activity of sitagliptin in an experimental T2D and the resulting acceptable pharmacokinetic profile, allow us to recommend the ZB-16 compound for further research.

## Author contributions

All authors contributed to the study's conception and design. MC and RK carried out the chemical synthesis and *in vitro* studies. IT, DK, EV, EM, and DB performed the *in vivo* analysis. All authors were involved in the analysis and interpretation of data, and each contributed to critical revision of the manuscript. All authors provided final approval of the version to be published.

### Conflict of interest statement

The authors declare that the research was conducted in the absence of any commercial or financial relationships that could be construed as a potential conflict of interest.

## Abbreviations

ADME: Absorption, Distribution, Metabolism and Excretion
DPP-4: Dipeptidyl peptidase-4
GLP-1: Glucagon-like peptide-1
GPR119: G protein-coupled receptor 119
T2D: type 2 diabetes mellitus
STZ-NA: streptozotocin-nicotinamide.
