# Supplementary material for: Chemistry and Hypoglycemic Activity of GPR119 Agonist ZB-16
Source: Front Endocrinol (Lausanne). 2018 Sep 19;9:543. doi: 10.3389/fendo.2018.00543 (PMC6156125; doi:10.3389/fendo.2018.00543)
Supplement: Supplementary file 1 [file Data_Sheet_1.docx]

Supplementary Material

Chemistry and hypoglycemic activity of novel GPR119 agonist ZB-16

I.N. Tyurenkov, D.V. Kurkin, D.A. Bakulin*, E.V. Volotova, E.I. Morkovin, M.A. Chafeev, R.N. Karapetian

*** Correspondence:** Corresponding Author: [mbfdoc@gmail.com](mailto:mbfdoc@gmail.com)

Supplement 1. Structures and activities of synthesized compounds

 GPR119 agonistic activity of test compounds

| **ID** | **Structure** | **Maximum tested concentration, μM** | **ЕC_50_, M** |
| --- | --- | --- | --- |
| C301-5917 |  | 30 | 2.74*10^-6^ |
| C530-0289 |  | 30 | 1.17*10^-5^ |
| C530-0291 |  | 30 | 2.42*10^-6^ |
| C530-0306 |  | 30 | 1.94*10^-5^ |
| C530-0320 |  | 30 | 2.20*10^-6^ |
| C530-0339 |  | 30 | 1.07*10^-5^ |
| C530-0358 |  | 30 | 3.10*10^-6^ |
| C530-0389 |  | 30 | 3.30*10^-6^ |
| D216-0545 |  | 30 | 2.75*10^-6^ |
| D469-0068 |  | 30 | 4.31*10^-7^ |
| K205-1693 |  | 30 | 5.37*10^-7^ |
| C301-5947A |  | 30 | 5.87*10^-7^ |
| C530-0315 |  | 30 | 7.13*10^-8^ |
| C530-0335 |  | 30 | 6.03*10^-7^ |
| C530-0386 |  | 30 | 7.58*10^-7^ |
| ZB-10 |  | 30 | 4.22*10^-7^ |
| ZB-12 |  | 30 | 4.05*10^-6^ |
| ZB-14 |  | 30 | 4.49*10^-7^ |
