# Supplementary material for: Chemistry and Hypoglycemic Activity of GPR119 Agonist ZB-16
Source: Front Endocrinol (Lausanne). 2018 Sep 19;9:543. doi: 10.3389/fendo.2018.00543 (PMC6156125; doi:10.3389/fendo.2018.00543)
Supplement: Supplementary file 2 [file Data_Sheet_2.docx]

Supplementary Material

Chemistry and hypoglycemic activity of novel GPR119 agonist ZB-16

I.N. Tyurenkov, D.V. Kurkin, D.A. Bakulin*, E.V. Volotova, E.I. Morkovin, M.A. Chafeev, R.N. Karapetian

*** Correspondence:** Corresponding Author: [mbfdoc@gmail.com](mailto:mbfdoc@gmail.com)

**Supplement 2. Formulas of non-active compounds**

Non-active compounds

| **ID/Structure** | **ID/Structure** |
| --- | --- |
|   C30-0277 |   C530-0283 |
|   C301-5916 |   C530-0276 |
|   C301-5918 |   C530-0271 |
|   С530-0265 |   C530-0267 |
|   C530-0292 |   C530-0310 |
|   C530-0297 |   C530-0323 |
|   C530-0298 |   C530-0324 |
|   C530-0300 |   C530-0331 |
|   C530-0334 |   K788-8629 |
|   C530-0366 |   K788-9236 |
|   C530-0369 |   ZB-04 |
|   C530-0379 | ZB-06 |
|   ZB-07 |   ZB-11 |
|   ZB-08 |  |
