# Supplementary material for: Chemistry and Hypoglycemic Activity of GPR119 Agonist ZB-16
Source: Front Endocrinol (Lausanne). 2018 Sep 19;9:543. doi: 10.3389/fendo.2018.00543 (PMC6156125; doi:10.3389/fendo.2018.00543)
Supplement: Supplementary file 3 [file Data_Sheet_3.docx]

Supplementary Material

Chemistry and hypoglycemic activity of novel GPR119 agonist ZB-16

I.N. Tyurenkov, D.V. Kurkin, D.A. Bakulin*, E.V. Volotova, E.I. Morkovin, M.A. Chafeev, R.N. Karapetian

*** Correspondence:** Corresponding Author: [mbfdoc@gmail.com](mailto:mbfdoc@gmail.com)


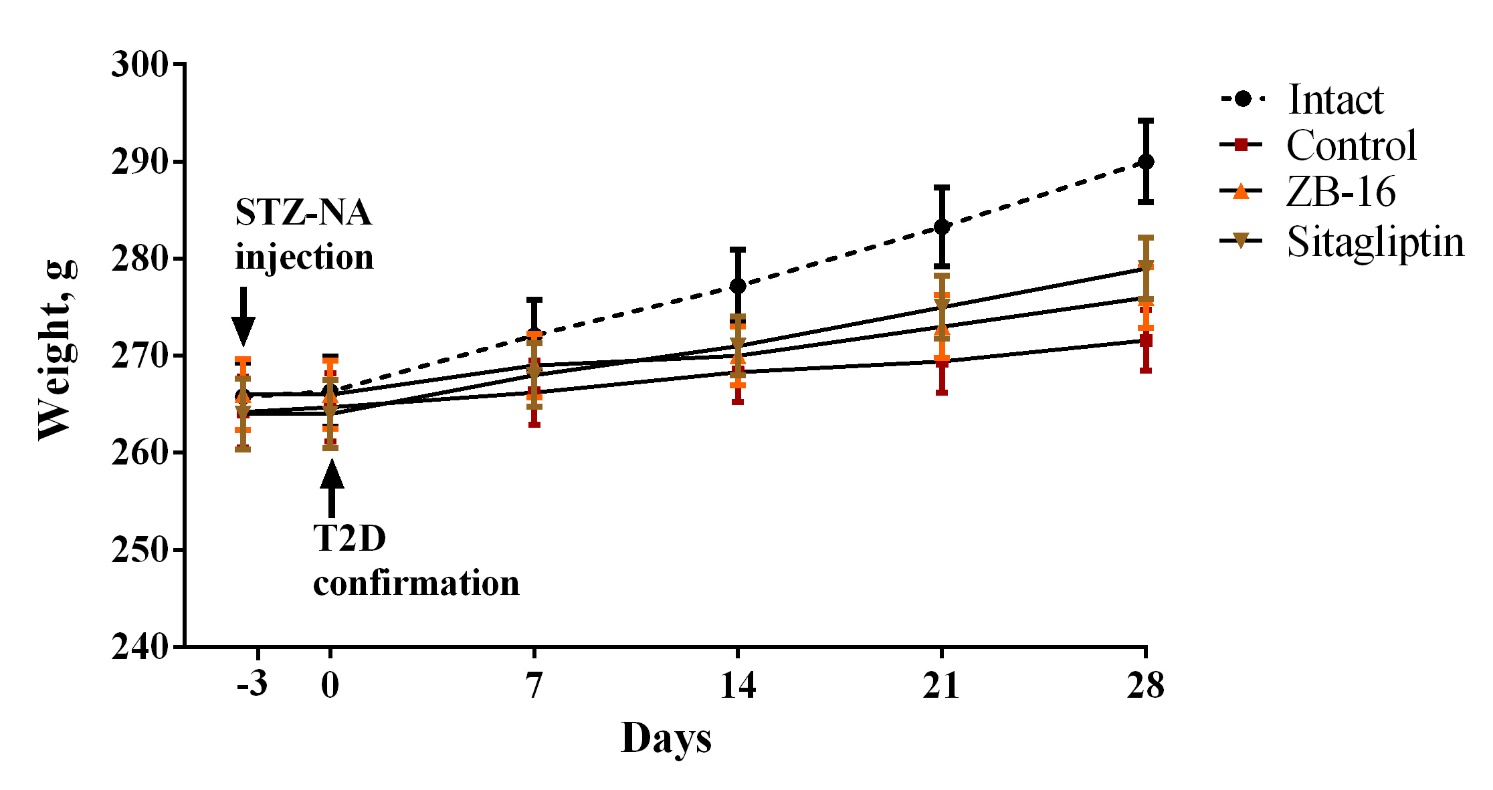
 **Supplement 3. Body weight of rats with streptozotocin-nicotinamide induced type 2 diabetes mellitus (STZ-NA T2D) for 28 days of treatment**

*Commentary:* The body weight tended to decrease in control group, but not in the treated groups; the increase was found in healthy animals, but there were no significant changes (p > 0.05 for all groups).
